# Understanding and Tuning Singlet–Triplet (S_1_–T_1_) Energy Gaps in Planar Organic Chromophores

**DOI:** 10.1002/anie.202502485

**Published:** 2025-04-07

**Authors:** Weixuan Zeng, Cheng Zhong, Hugo Bronstein, Felix Plasser

**Affiliations:** ^1^ Department of Chemistry University of Cambridge Cambridge CB2 1EW UK; ^2^ Hubei Key Lab on Organic and Polymeric Opto‐Electronic Materials, Sauvage Center for Molecular Sciences Department of Chemistry Wuhan University Wuhan 430072 China; ^3^ Cavendish Laboratory University of Cambridge Cambridge CB3 0HE UK; ^4^ Department of Chemistry Loughborough University Loughborough LE11 3TU UK

**Keywords:** Excited states, Organic chromophores, Quantum chemistry, Singlet–triplet energy gap

## Abstract

Molecules with large gaps between their first singlet and triplet excited states (Δ*E*_ST_) are key components of various modern technologies, most prominently singlet fission photovoltaics and triplet–triplet annihilation upconversion (TTA‐UC). The design of these molecules is hampered by the fact that only limited rules for maximizing Δ*E*_ST_ exist, other than increasing the overlap between the frontier molecular orbitals (FMO). Here we suggest a new strategy for tuning and maximizing Δ*E*_ST_ based on a detailed analysis of the underlying quantum mechanical energy terms. We present a model based on the transition density and derive three straightforward design rules: Δ*E*_ST_ values can be maximized by (i) minimizing the overall number of π‐electrons, (ii) reducing delocalization, and (iii) optimizing specific geometric interactions. The validity of these rules is first exemplified for a set of 18 hydrocarbon backbones before proceeding to a varied set of dye molecules, highlighting their transferability to realistic settings. We believe that the developed rules will provide an enormous boost to the field, enabling rational design instead of trial‐and‐error screening. More generally, this work demonstrates the power of going beyond the FMO approximation in designing advanced molecular materials.

## Graphical abstract

A new framework for tuning singlet–triplet (S_1_–T_1_) gaps based on electrostatic interactions of the transition density is developed. Three widely applicable design rules for maximizing the singlet–triplet gap are derived: minimizing the number of electrons, localizing the excitation, and optimizing through‐space interactions.

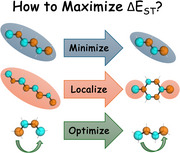

## Introduction

The energy gap between the first excited singlet (S_1_) and triplet (T_1_) states (Δ*E*_ST_) of an organic chromophore plays a critical role in dictating its photophysical properties and consequently its performance in a wide range of optoelectronic applications. The need for large Δ*E*_ST_ materials is prominently exemplified in the cases of singlet fission (SF)^[^ ^1^, ^2^, ^3^, ^4^ ^]^ and triplet–triplet annihilation upconversion (TTA‐UC),^[^ ^5^, ^6^ ^]^ both of which are promising technologies for improving solar cell efficiency.^[^ ^7^ ^]^ Reduced Δ*E*_ST_ values are needed for thermally activated delayed fluorescence (TADF).^[^ ^8^, ^9^ ^]^ It is by now well understood how to reduce Δ*E*_ST_ values via minimization of the spatial overlap between the frontier molecular orbitals (FMOs), and application of this strategy has led to the development of a vast number of TADF emitters.^[^ ^10^, ^11^ ^]^ Inverting this strategy, that is, maximizing the FMO overlap, does not by itself produce molecules with large enough Δ*E*_ST_ values for SF and TTA‐UC applications, and no alternative strategies that can be applied without excessive computational effort are available, leaving a gap in our ability to design high Δ*E*_ST_ materials.

This lack of ability to design high Δ*E*_ST_ materials holds back a number of crucial modern technologies. Most prominently, singlet fission has the potential to increase the performance of silicon solar cells beyond the Schockley–Queisser limit through the splitting of high‐energy photons into two lower‐energy charge‐carrier pairs. Practical materials require the condition of E(S_1_) > 2E(T_1_) to make the fission process exothermic, but also that T_1 _> 1.1 eV (the bandgap of Silicon) so that efficient sensitization can occur. Conversely for TTA, it has been suggested that the energies of T_1_ and S_1_ should lie around 1 and 1.9 eV, respectively. Other applications of materials profiting from large Δ*E*_ST_ values include new stable deep blue organic light‐emitting diodes (OLEDs), SF‐OLEDs, and TTA‐UC photocatalysts,^[^ ^12^, ^13^ ^]^ each implying further constraints on the required energy levels and other materials properties. It is abundantly clear that for their realization, absolute control over the Δ*E*_ST_ energy gap must be achieved. Despite decades of research, there is no easily actionable design rule to manipulate the S_1_–T_1_ energy gap, as evidenced by the almost complete absence of materials with appropriate energy levels for SF‐sensitized silicon solar cells, or the fact that most TTA‐UC materials are derivatives of a few basic polycyclic hydrocarbons.^[^ ^5^, ^6^, ^7^ ^]^

The topic of maximizing Δ*E*_ST_ values is an exciting and rapidly developing area of investigation and debate.^[^ ^5^, ^14^, ^15^ ^]^ Current design rules and searches for wide Δ*E*_ST_ materials have centered around identifying materials with enhanced diradical character or by tuning the gap between the highest occupied molecular orbital (HOMO) and the lowest unoccupied molecular orbital (LUMO) in already known wide Δ*E*_ST_ materials such that the energetic criteria for SF or TTA can be achieved.^[^ ^16^, ^17^, ^18^ ^]^ More recently, excited‐state aromaticity has been investigated as a promising alternative design route.^[^ ^15^, ^19^, ^20^ ^]^ These strategies have been successful at identifying new materials that can undergo these photophysical processes, and a great deal of new photophysical insight has resulted from these studies. However, new constraints come into play for real‐world applications, and it becomes vital to increase the design space of molecules to be studied.

Proceeding to a more mechanistic quantum mechanical picture, we note that within a two‐orbital two‐electron model (TOTEM),^[^ ^21^ ^]^ the Δ*E*_ST_ value is given as twice the exchange integral between the HOMO and the LUMO.^[^ ^22^, ^23^ ^]^ The exchange integral, in turn, can be reduced by minimizing the HOMO/LUMO overlap through spatial separation and/or deplanarization. These ideas give rise to readily applicable design guidelines that can be qualitatively applied with minimal computation. Conversely, there is no obvious FMO‐based strategy for generating materials with sufficiently large Δ*E*_ST_ values as required for SF or TTA‐UC. Therefore, the successful design of materials for these applications will arguably require transcending the FMO picture and proceeding to a more comprehensive understanding of the underlying quantum mechanical energy terms. Successful attempts at developing design guidelines beyond a straightforward HOMO/LUMO picture are rare^[^ ^24^, ^25^ ^]^ and certainly not applicable to S_1_–T_1_ energy gaps. On a more technical level, it was shown by Becke that, due to electron correlation, the observed singlet–triplet gaps in various hydrocarbons and heteroaromatics are only half of what would be expected from the simple HOMO/LUMO picture.^[^ ^26^ ^]^ Preliminary work by one of us has shown how the transition density provides a route toward a post‐MO understanding^[^ ^23^ ^]^ of singlet and triplet energies along with providing a meaningful decomposition of the contributing energy terms into Coulomb and exchange terms.^[^ ^27^, ^28^ ^]^

It is the purpose of this work to go further and to significantly push the boundaries of what can be achieved with pen‐and‐paper molecular materials design founded in chemical intuition, as applied to the case of Δ*E*_ST_ values. We show how our formalism can be translated into actionable design rules for generating wide Δ*E*_ST_ materials. We present an intuitive way of approximating the Δ*E*_ST_ value based on the spatial extent of and patterns found within the transition density and verify our model via time‐dependent density functional theory (TDDFT) computations. Studying, first, polyene and polycyclic aromatic hydrocarbon (PAH) building blocks, we highlight how the length, configuration, conformation of the backbone, and the formation of conjugated fused rings can all have significant effects on Δ*E*_ST_. We proceed to a wider discussion of singlet fission chromophores, built on these backbones, highlighting the power of rationalizing chromophore properties based on their backbones.

## Results and Discussion

**Scheme 1:**
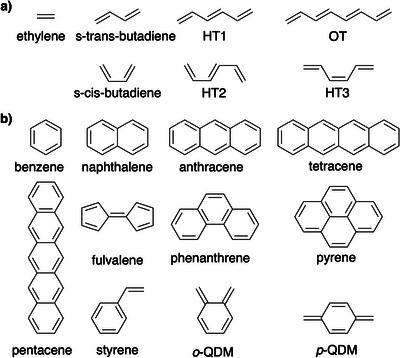
Conjugated hydrocarbon backbones investigated in this study: a) linear polyenes; b) polycyclic aromatic hydrocarbons.

A test set of 18 conjugated hydrocarbons, as shown in Scheme 1, was chosen to investigate the effect of delocalization and molecular geometry on Δ*E*_ST_. Importantly, this set of molecules represents the conjugated backbones of essentially every motif that is commonly used in organic chromophores. Computations on these molecules were performed at the TDDFT/M06‐2X level of theory;^[^ ^29^, ^30^ ^]^ the wave functions produced were analyzed using the libwfa library within Q‐Chem.^[^ ^31^, ^32^ ^]^ Δ*E*_ST_ values on this set of systems were evaluated by comparing the respective energies of the lowest singlet and triplet excited states, each with predominant HOMO–LUMO character (see Table S1). The reported values are vertical S_1_–T_1_ energy gaps, allowing us to study the direct electronic contributions to Δ*E*_ST_. This study does not further consider differential relaxation contributions that might affect Δ*E*_ST_, noting that these are (i) expected to be small and (ii) can be controlled by other means, such as rigidifying the backbone. Before proceeding, we also note that the M06‐2X energies used here are in very good agreement with a higher‐level coupled cluster reference^[^ ^33^, ^34^ ^]^ (see Figure S3), underscoring the reliability of the computational results.

**Figure 1:**
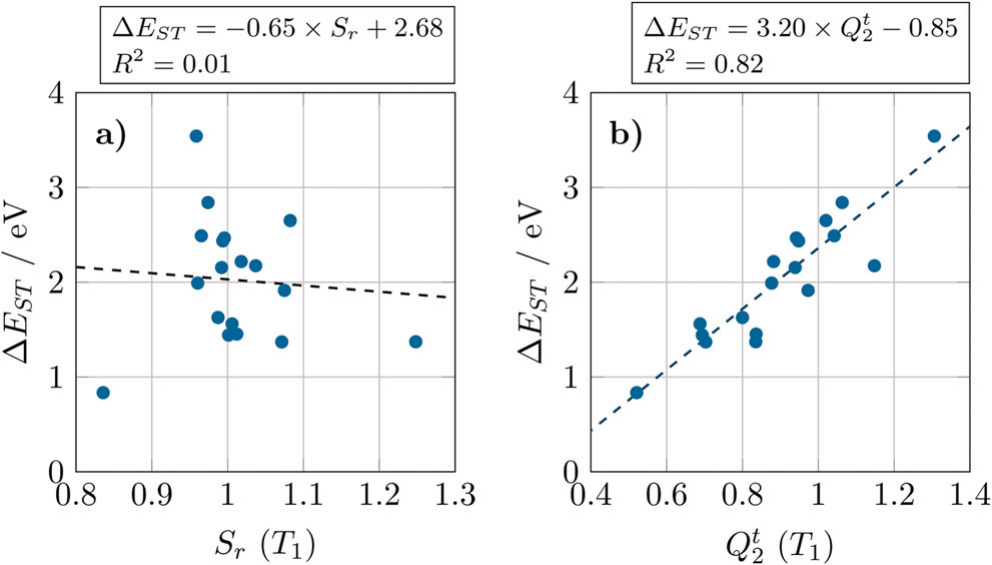
Comparison of a) the *S*_r_ index and b) the sum over squared transition charges ($Q_{2}^{t}$) in terms of their ability to predict S_1_–T_1_ gaps. Regression lines are shown as dashed lines; parameters of the fit are given in the top panel.

The Δ*E*_ST_ values thus evaluated range from 0.83 eV (fulvalene) up to 3.54 eV (ethylene). First, we were interested in whether the commonly used^[^ ^35^, ^36^ ^]^ *S*_r_ index, measuring the spatial overlap of the densities of the excitation hole and the excited electron,^[^ ^37^ ^]^ could serve as an indicator for this strong variation in Δ*E*_ST_. A scatter plot of *S*_r_ and Δ*E*_ST_ is shown in Figure 1a. Almost no correlation is visible with most points scattered at *S*_r_ values between 0.9 and 1.1 spanning S_1_–T_1_ gaps between 1.3 and 3.5 eV. The only successful example is shown on the lower left in Figure 1a: fulvalene, the only molecule in our dataset with a Δ*E*_ST_ value below 1 eV, is also the molecule with the lowest *S*_r_ value (0.84). Within our dataset, fulvalene is distinguished as the only non‐alternant hydrocarbon (possessing a five‐membered ring). Thus, at this point, we can say that alternant hydrocarbons are better candidates for high Δ*E*_ST_ materials following symmetry relations between HOMO and LUMO (cf. Ref. [38]). However, clearly, a more detailed model is needed to explain the strong variations in Δ*E*_ST_ within the alternant hydrocarbons.

**Figure 2:**
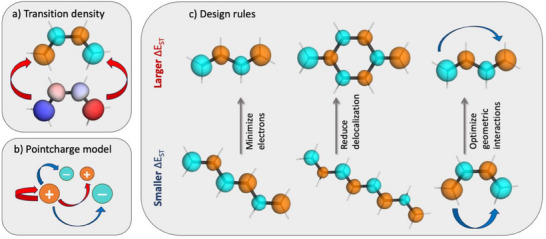
Summary of the developed design strategy bridging from quantum mechanical energy terms to practical rules: a) The singlet–triplet gap is computed via the transition density self‐repulsion as illustrated via the transition density (top) and its ESP (bottom); b) this is approximated via a point‐charge model to determine individual atomic contributions; c) design rules are developed for modulating Δ*E*_ST_. Repulsive interactions are marked as red arrows and attractive interactions as blue arrows.

Realizing that *S*_r_ is not suitable, we proceed to a different approach based on a more detailed consideration of the underlying energy terms. A summary of the developed design strategy is shown in Figure 2. Our strategy starts by noting that the S_1_–T_1_ gap can be associated with the self‐repulsion of the transition density (TD, ρ_*t*_) considered as a charge distribution in space.^[^ ^23^ ^]^ This fact is represented in Figure 2a by showing the TD along with its electrostatic potential. In the next step, we perform a population analysis of the TD to compute the transition charges. As highlighted in Figure 2b, the overall TD repulsion can thus be divided into intra‐atomic repulsion terms along with interatomic terms that may be either attractive or repulsive. At this point we can anticipate that Δ*E*_ST_ values will be maximal for small molecules and enhanced localization following the intramolecular terms, while also geometric interactions should play a role depending on how far apart the different point charges are in space.

We next develop a quantitative approach to evaluate the validity of our model via realistic calculations. As presented in Section S1, we obtain the working equation for the singlet–triplet gaps:

(1) $$\Delta E_{\mathrm{ST}}=UQ_{2}^{t}+V_{\mathrm{inter}}$$

Here $Q_{2}^{t}$ is the sum over the squares of the transition charges on the individual atoms. *U* is a global parameter representing the on‐site repulsion of two electrons residing on the same atom, in analogy to the Hubbard parameter commonly used in Pariser–Parr–Pople (PPP) theory.^[^ ^39^, ^40^ ^]^ *V*_inter_ collects all interatomic terms. The first term can be further divided, yielding:

(2) $$\Delta E_{\mathrm{ST}}=U\frac{Q_{a}^{t}\times Q_{a}^{t}}{n_{\mathrm{PR}}}+V_{\mathrm{inter}}$$

Here $Q_{a}^{t}$ is the sum over the absolute values of the transition charges,^[^ ^41^ ^]^ representing the overall amount of transition density present. *n*_PR_, defined as ${\left(Q_{a}^{t}\right)}^{2}/Q_{2}^{t}$, counts the number of atoms over which the TD is distributed, thus measuring delocalization. We note that $Q_{2}^{t}$ and $Q_{a}^{t}$ can be evaluated based on a simple population analysis, whereas an accurate evaluation of *V*_inter_ requires the two‐electron repulsion integrals. We implemented routines for the evaluation of the $Q_{2}^{t}$ and $Q_{a}^{t}$ terms from quantum chemistry computations within our wave function analysis library libwfa,^[^ ^31^ ^]^ whereas we will estimate *V*_inter_ as a residual in the fitting procedure.

To evaluate the validity of our model, we now feed Equation (1) with data from our TDDFT computations, and we plot $Q_{2}^{t}$ against the Δ*E*_ST_ values in Figure 1b. Already at this level (i.e., ignoring variations in *V*_inter_), a good correlation is obtained with an *R* ^2^ value of 0.82. The fit yields a value of 3.20 eV for the effective *U* parameter, which is about half the value typically used for PPP computations,^[^ ^39^ ^]^ but clearly represents the same physics. Within our fit, a constant term of −0.85 eV is obtained, which represents an averaged *V*_inter_ term. There is a good correlation along the whole range of values considered with all data points scattered around the line of best fit. The biggest overprediction for Δ*E*_ST_ values (+0.65 eV) is seen for benzene. The biggest underpredictions (both −0.30 eV) are seen for s‐*trans*‐butadiene and the all‐*trans* hexatriene isomer (**HT1**). As will be discussed in more detail below, this deviation can be used to estimate the *V_inter_* term highlighting that this term is particularly unfavorable for benzene, thus lowering Δ*E*_ST_, and favorable for the all‐*trans* polyenes.

Having verified the general validity of our model, we now proceed to its practical implications, and we sketch three practical design guidelines in Figure 2c. According to the right‐hand side of Equation (2), Δ*E*_ST_ is seen to be affected by three individual contributions: *n*_PR_, *V*_inter_, and $Q_{a}^{t}$. Crucially, the number of atoms involved (*n*_PR_) appears in the denominator, meaning that a reduction of *n*_PR_ can be used to increase Δ*E*_ST_. There are two possible strategies to do so: First, *n*_PR_ can be lowered by minimizing the overall number of electrons, for example, by moving from hexatriene to butadiene, as shown in Figure 2c (left). Second, *n*_PR_ can be lowered by reducing delocalization and, thus, confining the excitation to a smaller number of atoms. This is represented in Figure 2c (center) by comparing all‐*trans* octatetraene (**OT**) with ***p*‐QDM**. In the former case, the excitation is distributed over all atoms, while it is distinctly localized on the CH_2_ groups in the latter. Moreover, the interatomic potential *V*_inter_ can be tuned by optimizing the arrangement of the atoms in space. This is most clearly illustrated by comparing s‐*trans* and s‐*cis*‐butadiene, where the latter's Δ*E*_ST_ value is significantly reduced due to an attractive 1,4‐interaction between the outer carbon atoms.

Finally, we note that Δ*E*_ST_ is also affected by the total amount of charge ($Q_{a}^{t}$) of the transition density. The physical significance of this quantity has recently been discussed in Ref. [41], highlighting its connection to the ionicity of the state as understood within valence‐bond theory. However, as opposed to *n*_PR_ and *V*_inter_, we could not find an obvious way of using $Q_{a}^{t}$ for the purpose of molecular design and will, therefore, not discuss it further.

**Figure 3:**
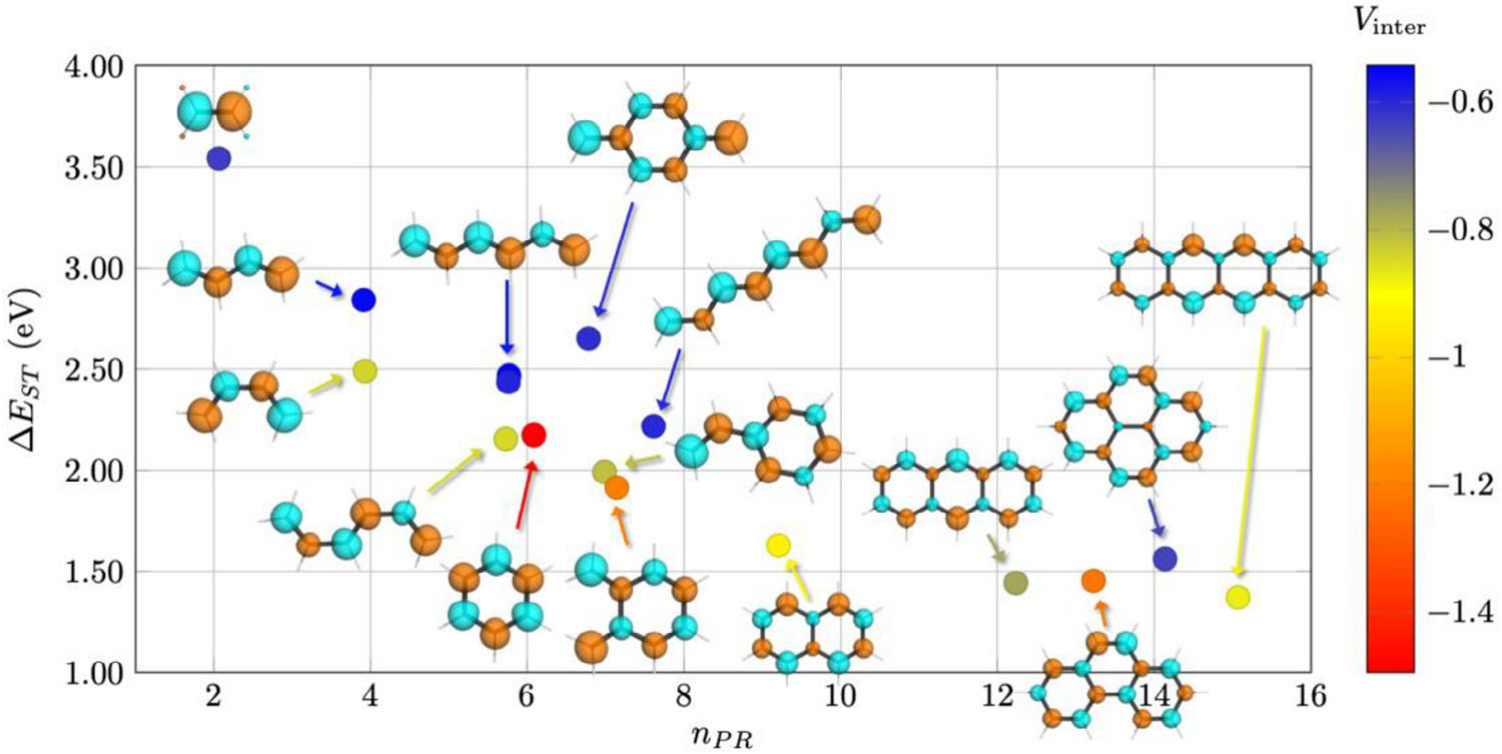
Singlet–triplet gaps of backbones plotted against the delocalization length (*n*_PR_). Points are color‐coded by the magnitude of the estimated interatomic interaction energy (*V*_inter_). Transition densities computed for T_1_ at the TDDFT/M06‐2X level are shown as inset.

Having developed a qualitative model, we now want to evaluate its effect quantitatively using realistic computations. For this purpose, we plot the Δ*E*_ST_ values against the delocalization length (*n*_PR_) as shown in Figure 3. The estimated value of the geometric interaction term (*V*_inter_) is represented via the color‐coding. The figures present results on all backbones except for fulvalene and pentacene, which are outside the presented range. In line with the previous discussion, we find a steady decrease of Δ*E*_ST_ with increasing *n*_PR_, starting from ethylene (3.54 eV) and going to the biggest molecule shown here, tetracene (1.37 eV). The high Δ*E*_ST_ value of ethylene can be explained by its small number of electrons (*n*_PR_ = 2.1) as well as the absence of non‐trivial stabilizing geometric interactions (aside from an attractive 1,2‐interaction, which is present in all molecules) yielding a *V*_inter_ value of −0.63 eV. Following ethylene, shown as blue and yellow dots, there are s‐*trans* and s‐*cis* butadiene. Their Δ*E*_ST_ values (2.84 vs. 2.49 eV) differ remarkably despite them being isomeric structures and having virtually identical *n*_PR_ and $Q_{a}^{t}$ values. The difference between them is ascribed to the *V*_inter_ term (−0.56 vs. −0.84 eV), thus highlighting the importance of geometric interactions and specifically the 1,4‐interactions illustrated in Figure 2c (right).

The linear hexatrienes (**HT1**‐**HT3**), shown next, also span a remarkable range in Δ*E*_ST_ values (2.16–2.47 eV), where the highest value is obtained for all‐*trans* hexatriene (**HT1**) and the lowest for the isomer with two s–*cis* interactions (**HT2**), which are again differentiated by their *V*_inter_ terms (−0.54 vs. −0.85 eV). Benzene (formally cyclo‐hexatriene) is positioned near the lower end of the linear hexatrienes. Out of all molecules considered, benzene has the most negative *V*_inter_ value (−1.49 eV), following from the presence of six attractive 1,2‐interactions and three attractive 1,4‐interactions, explaining its low Δ*E*_ST_ value when compared to the linear hexatrienes. Interestingly, benzene has a significantly enhanced total charge ($Q_{a}^{t}$) of 2.64 (vs. ∼2.3 for the linear hextrienes), counterbalancing some of the effect of the enhanced *V*_inter_ term. The molecules containing eight carbon atoms are shown toward the middle of Figure 3. At the top of these is *para*‐quinodimethane (***p*‐QDM**), possessing a strikingly high Δ*E*_ST_ value of 2.65 eV, exceeding s‐*cis*‐butadiene and all hexatrienes. This high Δ*E*_ST_ value can be explained via localization as well as geometric interactions. The former can be seen by its reduced *n*_PR_ value of 6.78 (compared to 7.61 for all‐*trans* octatetraene). The latter is represented by its less negative *V*_inter_ value compared to ***o*‐QDM** (−0.61 vs. −1.20 eV), deriving from the fact that the CH_2_ groups, containing most of the transition density, are much further apart in space.

The PAHs are shown on the right‐hand side of Figure 3. It is noteworthy that despite their increasing size, all PAHs considered retain Δ*E*_ST_ values around 1.5 eV and that there is no notable decrease with molecular size as seen for the smaller molecules on the left. This trend is in part explained by increased localization. Looking at the polyacenes, we find that the transition density is evenly localized over all six atoms in benzene, whereas it becomes more concentrated for naphthalene (*n*_PR_ = 9.2, 10 atoms), anthracene (*n*_PR_ = 12.2, 14 atoms), and tetracene (*n*_PR_ = 15.1, 18 atoms). Moreover, the *V*_inter_ terms become less pronounced when moving from benzene to anthracene reflecting enhanced localization on the *zigzag* edges and a concomitant decrease in stabilizing 1,2‐interactions. Out of the larger molecules, pyrene is particularly interesting, possessing an increased Δ*E*_ST_ value due to its enhanced localization (*n*_PR_ = 14.1, 18 atoms) and a *V*_inter_ value (−0.64 eV) similar to the best linear molecules. Finally, we note that the remarkable performance of the PAHs is not explained by *n*_PR_ and *V*_inter_ alone, but that also an increase in the overall amount of charge $Q_{a}^{t}$ plays a role.

Figure 3 highlights the relations between the Δ*E*_ST_ values and the shape of the transition density as computed via TDDFT. In the next step, we were interested in whether we could skip the TDDFT step and predict the shape of the transition density qualitatively without any quantum chemical calculation, thus having a purely pen‐and‐paper approach. To do so, we formally construct the wave function of the excited state as a combination of resonance structures where, in each case, one double bond is broken into a radical pair. Furthermore, when possible, we will rank these structures according to energy by counting their Clar sextets. We will then qualitatively construct the transition density from the dominant resonance structures, following similar ideas used to predict the distribution of unpaired electrons in PAHs.^[^ ^17^, ^42^, ^43^, ^44^, ^45^ ^]^

**Figure 4:**
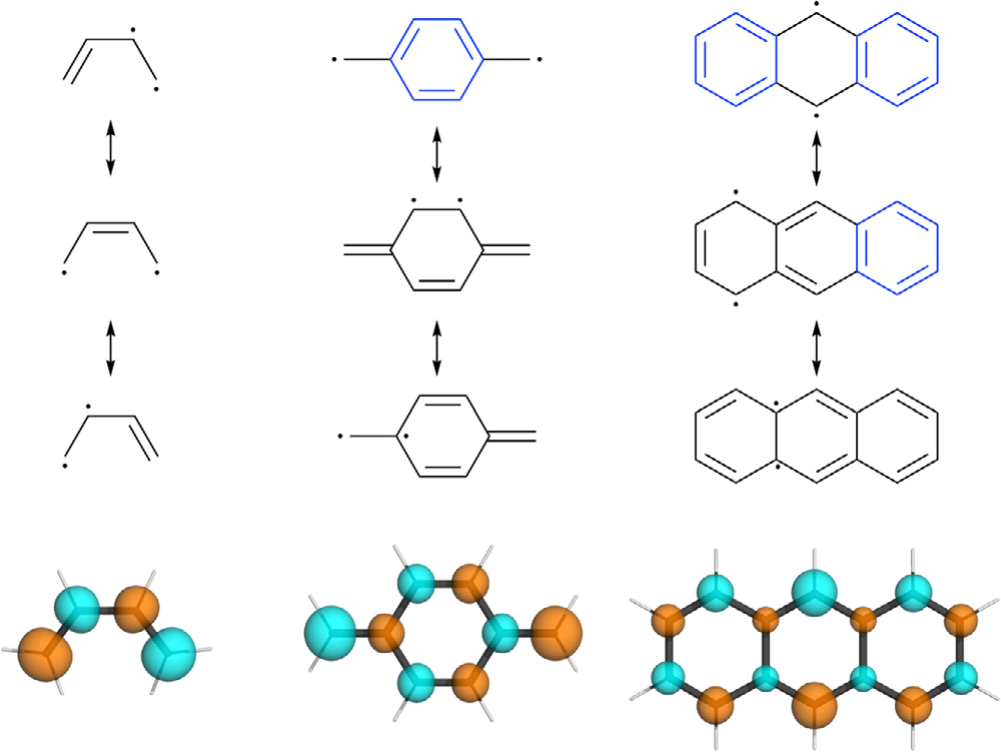
Rationalization of the shape of the T_1_ transition densities (bottom) via resonance structures (top) with Clar sextets highlighted in blue.

We analyze three selected molecules (s‐*cis*‐butadiene, ***p*‐QDM**, and anthracene), comparing resonance structures to transition density plots (Figure 4). The analysis of s‐*cis*‐butadiene is shown to the left in Figure 4. There are three possible ways of having a diradical along with an intact double bond: the diradical can either be on the right, the left, or the terminal carbon atoms. Viewing this, it is noteworthy that for each inner carbon atom, there is only one resonance structure with the radical located on it, whereas there are two each for the outer carbon atoms. Hence, one expects enhanced transition density on the outer carbons. This is indeed nicely seen for the transition density (shown on the bottom) and reflected by the transition charges, which are 0.60 *e* for an outer carbon atom and only 0.40 *e* for an inner one. Moving to ***p*‐QDM** (Figure 4, middle), we find that there is only one possible resonance structure that contains a Clar sextet, which is present if the radical centers are on the outer CH_2_ groups. Conversely, the aromaticity is broken whenever an unpaired electron is located on the benzene ring. Hence, we can anticipate that the unpaired electrons will be mostly localized on the outer carbon atoms. This is reflected in the transition density shown on the bottom and the enhanced transition charges on the outer carbon atoms (0.58 *e*) versus the inner ones (∼0.24 *e*). Anthracene is shown on the right as a representative of the polyacenes. In this case, it is noteworthy that only one resonance structure with two sextets exists, corresponding to the case where both unpaired electrons are localized on the atoms at the center of the *zigzag* edge. This central carbon atom is indeed the one with the largest transition density contribution (0.38 *e*). If the radical pair is moved to one of the outer rings, then one finds that one of the Clar sextet remains. By contrast, both sextets are lost if the radicals are moved to the inner carbon atoms. Indeed, one finds enhanced contributions on the outer carbon atoms (0.23 *e*, 0.19 *e*) compared to the inner ones (0.12 *e*). This effect is even more pronounced in pyrene (see Figure 3), where the inner carbon atoms have transition charges of only 0.09 *e*. More generally, these considerations can be extended to the whole polyacene series, explaining why unpaired electrons in these systems are localized on the zigzag edges,^[^ ^46^, ^47^ ^]^ and this localization provides a rationale for their enhanced Δ*E*_ST_ values.

Reviewing Figure 4, we note the crucial effects of pro‐aromaticity for localizing and stabilizing the radicals to ultimately increase Δ*E*_ST_ values. Here, pro‐aromaticity refers to the fact that the number of Clar sextets in these molecules can be increased when moving from a closed shell to a diradical resonance structure. Pro‐aromaticity and its consequences have been widely studied in ***p*‐QDM** and related molecules,^[^ ^43^, ^48^, ^49^, ^50^ ^]^ and it is certainly noteworthy that the remarkably high Δ*E*_ST_ value of polyacenes can be related to similar effects.

**Figure 5:**
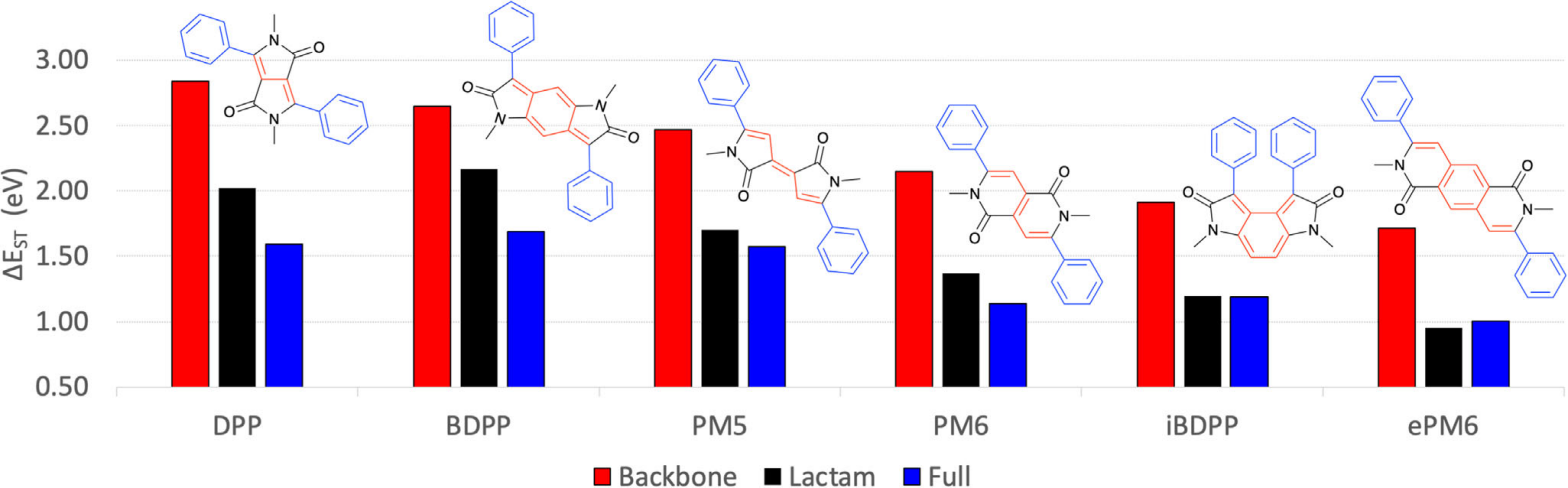
Analysis of diphenyl‐substituted lactams built from the backbones discussed above. Δ*E*_ST_ values are shown for just the backbone (red), the lactam dye (black), and the full diphenyl substituted molecule (blue).

The above understanding can be applied to a wider range of chromophores that essentially cover the entire spectrum of planar conjugated materials used in organic electronics. First, we will investigate a series of fused lactam chromophores (Figure 5), several of which have been shown to be of relevance to singlet fission design, including diketopyrrolopyrrole (**DPP**),^[^ ^51^, ^52^, ^53^ ^]^ two isomeric lactam analogues of Pechmann dyes (**PM5** and **PM6**),^[^ ^54^, ^55^, ^56^, ^57^ ^]^ and benzodipyrrolidone (**BDPP**).^[^ ^58^, ^59^ ^]^ In addition, we present computations on a hypothetical isomeric form of **BDPP** (denoted **iBDPP**) and an extended form of **PM6** (denoted **ePM6**). We would argue that prior to this work it would have been impossible to predict on paper which of these materials has the larger or smaller Δ*E*_ST_. Here we will show how to do so based on the knowledge gained above.

We start the discussion based on the underlying hydrocarbon backbone, shown in red in Figure 5. The Δ*E*_ST_ values for the backbones are shown as red bars, and the molecules are ordered from left to right according to decreasing Δ*E*_ST_ values. We know from earlier that shorter, *trans* polyene backbones have the highest Δ*E*_ST_ along with *pro*‐aromatic ***p*‐QDM** motifs with Δ*E*_ST_ decreasing with increasing numbers of double bonds and s‐*cis* linkages, and these trends are reflected in Figure 5. Proceeding to the larger molecules, we show the Δ*E*_ST_ values of the lactams and the final diphenyl substituted dyes as black and blue bars, respectively. In line with the above discussion, the enhanced delocalization introduced by adding atoms in this fashion goes along with a lowering in Δ*E*_ST_. The only partial exception to this rule is **ePM6**, where the final diphenyl substitution happens to have a slightly positive effect on Δ*E*_ST_. More importantly, also the trends among the substituted molecules are largely consistent with the bare backbones, meaning that qualitative arguments based on the backbone have predictive power regarding the final dye molecules. The first three molecules shown (**DPP**, **BDPP**, and **PM5**) retain Δ*E*_ST_ values above 1.5 eV, whereas Δ*E*_ST_ values are closer to 1.0 eV for the others, explaining why only the first class has been implied in the development of SF materials. To understand the differences in more detail, we can compare, for example, the isomeric diphenyl‐substituted **BDPP** and **iBDPP** molecules and trace their dramatic difference in Δ*E*_ST_ values (1.69 vs. 1.19 eV) back to their underlying ***p*‐QDM**/***o*‐QDM** motifs. A similar discussion can be made comparing **PM5** and **PM6** as well as their lactone versions.^[^ ^55^ ^]^ In summary, Figure 5 highlights the power of extrapolating from the properties of conjugated backbones to realistic dye molecules.

**Figure 6:**
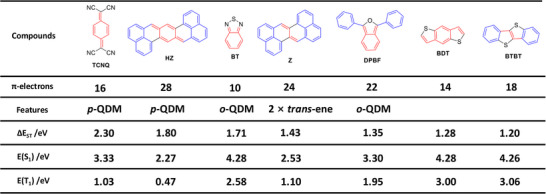
Analysis of π‐conjugated chromophores considering how their Δ*E*_ST_ values change with the number of π‐electrons and varying structural motifs.

Now, we examine a wider model pool with the key data collected in Table S3. Figure 6 shows the structures and calculated S_1_ and T_1_ energies of representative monomers or building blocks found throughout all aspects of organic electronics, including benzodithiophene (**BDT**) and benzothiadiazole (**BT**) for organic photovoltaics (**OPV**),^[^ ^60^ ^]^ [1]benzothieno[3,2‐b]benzothiophene (**BTBT**) for organic field‐effect transistor (OFET),^[^ ^61^ ^]^ 1,3‐diphenylisobenzofuran (**DPBF**),^[^ ^62^ ^]^ zethrene diradicaloids (**Z** and **HZ**)^[^ ^43^, ^63^ ^]^ and tetracyanoquinodimethane (**TCNQ**) for SF.^[^ ^2^, ^64^ ^]^

Within Figure 6, we present the expected dominant resonance structure for each molecule, using the rule that this should be a neutral closed‐shell resonance structure with a maximal number of disjoint Clar sextets (cf. Refs [17, 42, 43, 44, 45]). Even more simply than before, we now just consider how many π‐electrons the systems have and whether this dominant resonance structure has any features that promote large Δ*E*_ST_. We expect molecules with either a smaller number of π‐electrons or with s‐*trans*‐linkages/***p*‐QDM** motifs to have generally larger Δ*E*_ST_’s, and molecules with both can be expected to have the largest Δ*E*_ST._ On inspection of the materials pool, we find that the two molecules possessing ***p*‐QDM** motifs (**TCNQ** and **HZ**) also have the highest Δ*E*_ST_ values. **BT**, possessing the smallest number of π‐electrons, is ranked third. **Z** follows with a reasonably high Δ*E*_ST_ of 1.43 eV despite having 24 π‐electrons. The high value can be explained by viewing it as an s‐*trans*‐butadiene unit flanked by two naphthalenes. **DFBF**, possessing 22 π‐electrons (including the outer phenyl rings), follows with a reduced Δ*E*_ST_ of 1.35 eV. The final two molecules shown, **BDT** and **BTBT**, possess lower Δ*E*_ST_ values around 1.2 eV. These lower values can be explained by the fact that these molecules possess no special motifs for raising Δ*E*_ST_.

## Conclusion

We have presented practically applicable design guidelines for generating chromophores with maximal Δ*E*_ST_ values. Whereas consideration of HOMO/LUMO overlap integrals provides an effective approach to minimizing Δ*E*_ST_ values, it provides no actionable route for increasing Δ*E*_ST_ values of planar π‐conjugated systems. Here, we present a new model for tuning Δ*E*_ST_ values based on the spatial extent and shape of the transition density. The transition density does not only provide an intuitive basis for visualizing the contributions of individual atoms to Δ*E*_ST_, but crucially, it also allows for the development of straightforward design guidelines that can be applied without further computation. Three such rules emerge: Δ*E*_ST_ values can be maximized by (i) minimizing the number of π‐electrons, (ii) reducing delocalization, and (iii) optimizing geometric interactions. Two specific structural motifs are identified in light of these rules. Small all‐*trans* polyenes are favorable following rules (i) and (iii). More strikingly, the pro‐aromatic ***p*‐QDM** motif is selected following rules (ii) and (iii), and we highlight its ability to maintain substantial Δ*E*_ST_ values even in larger π‐conjugated systems. We show how, based on simple hydrocarbon backbones, a variety of lactam dyes can be built whose Δ*E*_ST_ values nicely follow our developed rules. Finally, we investigate a wider range of chromophores implied for SF, highlighting how the interplay between the number of π‐conjugated electrons and the presence of favorable structural motifs consistently affects the energetics of the materials studied. These examples show that the new models are applicable on three levels: in the context of quantitative TDDFT results, qualitative considerations of resonance structures, as well as phenomenological discussions of motifs and electron counts.

The new guidelines are expected to interact favorably with existing considerations of diradical character and excited‐state aromaticity.^[^ ^16^, ^18^, ^19^, ^65^ ^]^ Diradical character can be used to destabilize S_0_, excited‐state aromaticity to concomitantly stabilize T_1_ and S_1_ while the present rules allow to modulate the gap between T_1_ and S_1_. This provides three different levers for tuning excited energies in planar systems (see Ref. [55] as an initial example for exploiting the latter two).

In summary, we believe that the newly developed rules will provide an enormous boost to SF and related efforts of developing molecules with tuned Δ*E*_ST_. More generally, we believe that the presented approach can stimulate the wider molecular design field by showing the power of proceeding beyond the FMO picture and by highlighting how complicated quantum mechanical expressions can be simplified into actionable pen‐and‐paper design rules.

## Supporting Information

Details of the developed model (Section S1), computational details (Section S2), computational results (Tables S1–S3), and correlation of TDDFT and CC2 results (Figure S3) are given.

## Conflict of Interests

The authors declare no conflict of interest.

## Supporting information



Supporting Information

## Data Availability

The data that support the findings of this study are openly available in Loughborough University's research repository at DOI: 10.17028/rd.lboro.27646671.
