# Supplementary material for: Understanding and Tuning Singlet–Triplet (S1–T1) Energy Gaps in Planar Organic Chromophores
Source: Angew Chem Int Ed Engl. 2025 Apr 7;64(21):e202502485. doi: 10.1002/anie.202502485 (PMC12087868; doi:10.1002/anie.202502485)
Supplement: Supplementary file 1 — Supporting Information [file ANIE-64-e202502485-s001.pdf]

Understanding and Tuning Singlet-Triplet (S_1/T_1) Energy Gaps in Planar Organic Chromophores

Weixuan Zeng,[1] Cheng Zhong,[2] Hugo Bronstein,[1,3*] Felix Plasser[4*]

[1] Department of Chemistry, University of Cambridge, Cambridge, CB2 1EW, UK

[2] Hubei Key Lab on Organic and Polymeric Opto-Electronic Materials, Sauvage Center for Molecular Sciences, Department of Chemistry, Wuhan University, Wuhan, 430072, China

[3] Cavendish Laboratory, University of Cambridge, Cambridge, CB3 0HE, UK

[4] Department of Chemistry, Loughborough University, Loughborough, LE11 3TU, UK

SECTION S1: DETAILS OF THE DEVELOPED MODEL

Here, we present the mathematical background of the developed model. We start by realizing that, assuming no changes in the CI vectors, the singlet-triplet gap (ΔE_{ST}) is determined by the Coulomb interaction of the transition density (TD) with respect to itself or, alternatively, as an overlap between the TD and its ESP (V_t).

$$\Delta E_{ST} = \iint \frac{\rho_t(r_1)\rho_t(r_2)}{r_{12}} dr_1 dr_2 = \int \rho_t(r_1) \left(\int \frac{\rho_t(r_2)}{r_{12}} dr_2 \right) dr_1 = \int \rho_t(r_1)\, V_t(r_1) dr_1 \tag{1}$$

This is represented pictorially in Figure 2a of the main text. We, next, divide the TD into atomic contributions noting that the charge on every atom reacts with itself as well as all the other atoms. The interaction with itself and charges of the same sign is repulsive (raising ΔE_{ST}); the interaction of opposite charges is attractive (lowering ΔE_{ST}). For practical computations the atomic contributions are computed via a population analysis of the TD, yielding the so-called transition charges (denoted q_A).

The transition density repulsion can now be divided into a sum of intra and interatomic contributions. The intraatomic contributions are approximated as

$$\mathrm{V}_{intra} \approx U \sum_A {q_A}^2 \tag{2}$$

where the sum goes over all atoms A of the system. The parameter U represents the interelectronic repulsion of two electrons residing on the same atom. Using the definition $Q_2^t = \sum_A {q_A}^2$, the intraatomic term can be rewritten as

$$\mathrm{V}_{intra} \approx U Q_2^t \tag{3}$$

highlighting that this term is proportional to the electronic-structure descriptor Q_2^t as implemented within our libwfa library. The term can be further divided into

$$V_{intra} \approx U \frac{Q_a^t \times Q_a^t}{n_{PR}} \tag{4}$$

as discussed in the main text. The interatomic term is initially approximated as

$$V_{inter} \approx \sum_{A \neq B} \frac{q_A q_B}{r_{AB}} \tag{5}$$

where r_{AB} is the spatial separation between atoms A and B. Equation (5) highlights how to maximize V_{inter}: charges of the same sign should be close together, charges of opposite sign should be far apart. However, we note that a practical evaluation of V_{inter} proved challenging. This would probably require an exact evaluation of the involved two-electron integrals along with appropriate screening effects. Therefore, when discussing V_{inter} in the text, we do not compute it explicitly, but we simply assign all residual effects (not captured by V_{intra}) to this term noting that this already provides consistent and instructive results. Combining the intra- and interatomic terms yields the final working equation

$$\Delta E_{ST} \approx U \frac{Q_a^t \times Q_a^t}{n_{PR}} + V_{inter} \tag{6}$$

as discussed in the main text.

SECTION S2: COMPUTATIONAL DETAILS

Molecular structures were optimized at the B3LYP/def2-SVP [1–3] level of theory using Grimme's D3 dispersion correction.[4] TDDFT computations on backbones as well as lactams were carried out at the M06-2X/def2-SVP[3,5] level of theory as implemented in Q-Chem 6.1.[6] These computations employed full TDDFT (rather than the Tamm-Dancoff approximation). The Q_2^t and Q_a^t values were determined based on transition charges computed via a Löwdin partitioning of the transition density using the libwfa wave function analysis library.[7] The requisite analysis is activated within Q-Chem by simply setting the STATE_ANALYSIS = TRUE keyword.

TDDFT computations on the set of molecules presented in Fig. 6 were carried out at the M06-2X/def2-SVP level of theory as implemented in GAUSSIAN 16 package.[8]

Additional computations were performed at the RI-CC2 (second order approximate coupled cluster using the resolution of the identity approximation) level along with the def2-TZVPP basis set, as implemented in Turbomole 7.4.[9,10]

SECTION S3: COMPUTATIONAL DATA

Table S1: Computational data for backbones: states involved in the determination of the ΔE_{ST} values,[a] excitation energies (ΔE), oscillator strengths (f), and TD descriptors as defined above, all presented for the relevant singlet (S) and triplet (T) state.[b]

molecule	states	$\boldsymbol{\Delta E}$ (T)	$\boldsymbol{\Delta E}$ (S)	f (S)	$\boldsymbol{\Delta E_{ST}}$	$\boldsymbol{Q_a^t}$ (T)	$\boldsymbol{Q_2^t}$ (T)	$\boldsymbol{n_{PR}}$ (T)	$\boldsymbol{Q_a^t}$ (S)	$\boldsymbol{Q_2^t}$ (S)	$\boldsymbol{n_{PR}}$ (S)
ethene	T_1 / S_2	4.696	8.238	0.390	3.542	1.642	1.306	2.064	0.923	0.337	2.528
s-cis butadiene	T_1 / S_1	3.126	5.616	0.322	2.490	2.023	1.042	3.928	0.926	0.186	4.610
s-trans butadiene	T_1 / S_1	3.372	6.213	0.701	2.841	2.039	1.063	3.911	0.893	0.176	4.531
OT	T_1 / S_1	2.230	4.448	1.498	2.218	2.591	0.882	7.611	0.928	0.097	8.878
styrene	T_1 / S_2	3.411	5.402	0.341	1.991	2.475	0.877	6.985	1.005	0.122	8.279
anthracene	T_1 / S_1	2.170	3.612	0.085	1.442	2.914	0.694	12.235	0.955	0.071	12.845
benzene	T_1 / S_2	4.350	6.524	0.000	2.174	2.643	1.148	6.085	1.315	0.251	6.889
fulvalene	T_1 / S_1	1.931	2.766	0.004	0.835	2.049	0.521	8.058	0.608	0.042	8.802
naphthalene	T_1 / S_2	3.169	4.797	0.084	1.628	2.714	0.800	9.207	1.042	0.106	10.243
o-QDM	T_1 / S_1	1.532	3.446	0.147	1.914	2.636	0.973	7.141	0.812	0.087	7.579
HT1	T_1 / S_1	2.659	5.126	1.090	2.467	2.331	0.942	5.768	0.910	0.123	6.733
HT2	T_1 / S_1	2.345	4.500	0.547	2.155	2.319	0.939	5.727	0.970	0.137	6.868
HT3	T_1 / S_1	2.696	5.132	0.949	2.436	2.337	0.948	5.761	0.890	0.118	6.713
pentacene	T_1 / S_1	0.886	2.258	0.068	1.372	3.845	0.835	17.705	0.880	0.045	17.209
phenanthrene	T_1 / S_2	3.188	4.641	0.093	1.453	3.325	0.836	13.224	1.260	0.108	14.700
p-QDM	T_1 / S_1	1.927	4.578	0.852	2.651	2.630	1.020	6.781	0.736	0.105	5.159
pyrene	T_1 / S_2	2.489	4.050	0.343	1.561	3.119	0.688	14.140	1.109	0.083	14.818
tetracene	T_1 / S_1	1.446	2.816	0.077	1.370	3.255	0.703	15.071	0.911	0.054	15.369

[a] The ΔE_{ST} values were always computed with respect to T_1 as triplet state. In most cases S_1 was used for the singlet state, except for cases where S_1 possessed different character to T_1. Practically, we always chose the lowest singlet with a Q_a^t value above 0.5 to identify the appropriate ionic HOMO/LUMO state.

[b] The analysis presented in the article is always based on the T_1 transition charges and descriptors [Q_a^t (T), Q_2^t (T), and Q_2^t (T)]. This choice was made as singlet states showed σ-polarization in their TD unnecessarily complicating the analysis. The T_1 transition charges can be seen as the formal starting point for the singlet HOMO/LUMO state, which however includes enhanced σ-correlation to relieve exchange repulsion.

Table S2: Computational data for lactam dyes: states involved in the determination of the ΔE_{ST} values, excitation energies (ΔE), oscillator strengths (f). "Ph-X" refers to "X" with the added phenyl groups.

molecule	states	$\boldsymbol{\Delta E}$ (T)	$\boldsymbol{\Delta E}$ (S)	f (S)	$\boldsymbol{\Delta E_{ST}}$
DPP	T_1 / S_1	1.567	3.550	0.305	2.023
Ph-DPP	T_1 / S_1	1.334	2.919	0.458	1.586
BDPP	T_1 / S_2	1.587	3.761	0.772	2.174
Ph-BDPP	T_1 / S_2	1.250	2.936	0.817	1.686
PM5	T_1 / S_1	1.134	2.835	0.274	1.701
Ph-PM5	T_1 / S_1	0.940	2.506	0.798	1.566
PM6	T_1 / S_1	2.463	3.809	0.266	1.346
Ph-PM6	T_1 / S_1	2.021	3.156	0.773	1.136
iBDPP	T_1 / S_1	0.891	2.085	0.095	1.194
Ph-iBDPP	T_1 / S_1	0.928	2.114	0.097	1.186
ePM6	T_1 / S_1	2.887	3.837	0.108	0.950
Ph-ePM6	T_1 / S_1	2.705	3.708	0.388	1.003

Table S3: Computational data for general singlet fission materials: states involved in the determination of the ΔE_{ST} values, excitation energies (ΔE), oscillator strengths (f).

molecule	states	$\boldsymbol{\Delta E}$ (T)	$\boldsymbol{\Delta E}$ (S)	f (S)	$\boldsymbol{\Delta E_{ST}}$
TCNQ	T_1 / S_1	1.028	3.330	1.130	2.303
HZ	T_1 / S_1	0.473	2.268	1.055	1.795
BT	T_1 / S_1	2.578	4.284	0.069	1.706
Z	T_1 / S_1	1.097	2.530	0.661	1.433
DPBF	T_1 / S_1	1.948	3.300	0.532	1.351
BDT	T_1 / S_1	3.001	4.277	0.157	1.275
BTBT	T_1 / S_1	3.059	4.263	0.199	1.203

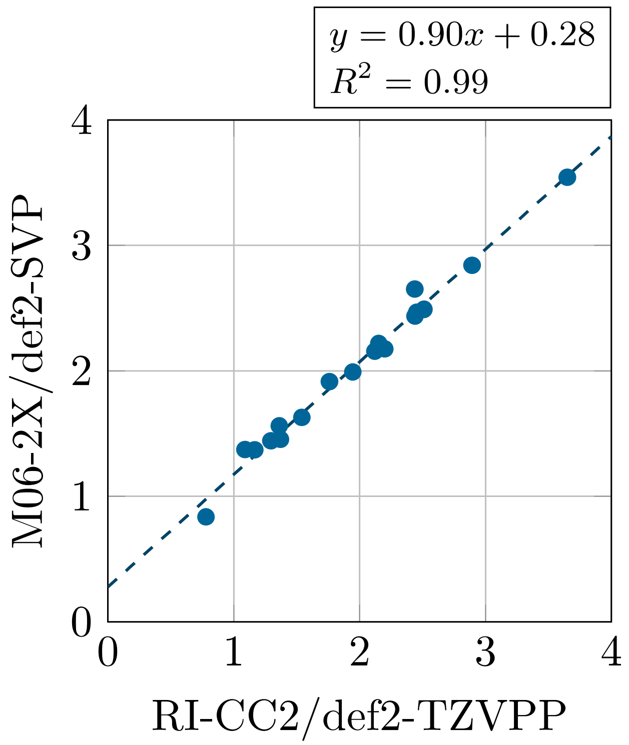

Figure S3: Correlation of singlet triplet gaps at the M06-2X/def2-SVP level (as used within this study) and the RI-CC2/def2-TZVPP reference.

References

(1) Becke, A. D. Density-functional Thermochemistry. III. The Role of Exact Exchange. *J Chem Phys* **1993**, *98* (7), 5648–5652. https://doi.org/10.1063/1.464913.

(2) Lee, C.; Yang, W.; Parr, R. G. Development of the Colle-Salvetti Correlation-Energy Formula into a Functional of the Electron Density. *Phys Rev B* **1988**, *37* (2), 785–789. https://doi.org/10.1103/PhysRevB.37.785.

(3) Weigend, F.; Ahlrichs, R. Balanced Basis Sets of Split Valence, Triple Zeta Valence and Quadruple Zeta Valence Quality for H to Rn: Design and Assessment of Accuracy. *Physical Chemistry Chemical Physics* **2005**, *7* (18), 3297. https://doi.org/10.1039/b508541a.

(4) Grimme, S.; Ehrlich, S.; Goerigk, L. Effect of the Damping Function in Dispersion Corrected Density Functional Theory. *J Comput Chem* **2011**, *32* (7), 1456–1465. https://doi.org/10.1002/jcc.21759.

(5) Zhao, Y.; Truhlar, D. G. The M06 Suite of Density Functionals for Main Group Thermochemistry, Thermochemical Kinetics, Noncovalent Interactions, Excited States, and Transition Elements: Two New Functionals and Systematic Testing of Four M06-Class Functionals and 12 Other Function. *Theor Chem Acc* **2008**, *120* (1–3), 215–241. https://doi.org/10.1007/s00214-007-0310-x.

(6) Epifanovsky, E.; Gilbert, A. T. B.; Feng, X.; Lee, J.; Mao, Y.; Mardirossian, N.; Pokhilko, P.; White, A. F.; Coons, M. P.; Dempwolff, A. L.; Gan, Z.; Hait, D.; Horn, P. R.; Jacobson, L. D.; Kaliman, I.; Kussmann, J.; Lange, A. W.; Lao, K. U.; Levine, D. S.; Liu, J.; McKenzie, S. C.; Morrison, A. F.; Nanda, K. D.; Plasser, F.; Rehn, D. R.; Vidal, M. L.; You, Z.-Q.; Zhu, Y.; Alam, B.; Albrecht, B. J.; Aldossary, A.; Alguire, E.; Andersen, J. H.; Athavale, V.; Barton, D.; Begam, K.; Behn, A.; Bellonzi, N.; Bernard, Y. A.; Berquist, E. J.; Burton, H. G. A.; Carreras, A.; Carter-Fenk, K.; Chakraborty, R.; Chien, A. D.; Closser, K. D.; Cofer-Shabica, V.; Dasgupta, S.; de Wergifosse, M.; Deng, J.; Diedenhofen, M.; Do, H.; Ehlert, S.; Fang, P.-T.; Fatehi, S.; Feng, Q.; Friedhoff, T.; Gayvert, J.; Ge, Q.; Gidofalvi, G.; Goldey, M.; Gomes, J.; González-Espinoza, C. E.; Gulania, S.; Gunina, A. O.; Hanson-Heine, M. W. D.; Harbach, P. H. P.; Hauser, A.; Herbst, M. F.; Hernández Vera, M.; Hodecker, M.; Holden, Z. C.; Houck, S.; Huang, X.; Hui, K.; Huynh, B. C.; Ivanov, M.; Jász, Á.; Ji, H.; Jiang, H.; Kaduk, B.; Kähler, S.; Khistyaev, K.; Kim, J.; Kis, G.; Klunzinger, P.; Koczor-Benda, Z.; Koh, J. H.; Kosenkov, D.; Koulias, L.; Kowalczyk, T.; Krauter, C. M.; Kue, K.; Kunitsa, A.; Kus, T.; Ladjánszki, I.; Landau, A.; Lawler, K. V.; Lefrancois, D.; Lehtola, S.; Li, R. R.; Li, Y.-P.; Liang, J.; Liebenthal, M.; Lin, H.-H.; Lin, Y.-S.; Liu, F.; Liu, K.-Y.; Loipersberger, M.; Luenser, A.; Manjanath, A.; Manohar, P.; Mansoor, E.; Manzer, S. F.; Mao, S.-P.; Marenich, A. V.; Markovich, T.; Mason, S.; Maurer, S. A.; McLaughlin, P. F.; Menger, M. F. S. J.; Mewes, J.-M.; Mewes, S. A.; Morgante, P.; Mullinax, J. W.; Oosterbaan, K. J.; Paran, G.; Paul, A. C.; Paul, S. K.; Pavošević, F.; Pei, Z.; Prager, S.; Proynov, E. I.; Rák, Á.; Ramos-Cordoba, E.; Rana, B.; Rask, A. E.; Rettig, A.; Richard, R. M.; Rob, F.; Rossomme, E.; Scheele, T.; Scheurer, M.; Schneider, M.; Sergueev, N.; Sharada, S. M.; Skomorowski, W.; Small, D. W.; Stein, C. J.; Su, Y.-C.; Sundstrom, E. J.; Tao, Z.; Thirman, J.; Tornai, G. J.; Tsuchimochi, T.; Tubman, N. M.; Veccham, S. P.; Vydrov, O.; Wenzel, J.; Witte, J.; Yamada, A.; Yao, K.; Yeganeh, S.; Yost, S. R.; Zech, A.; Zhang, I. Y.; Zhang, X.; Zhang, Y.; Zuev, D.; Aspuru-Guzik, A.; Bell, A. T.; Besley, N. A.; Bravaya, K. B.; Brooks, B. R.; Casanova, D.; Chai, J.-D.; Coriani, S.; Cramer, C. J.; Cserey, G.; DePrince, A. E.; DiStasio, R. A.; Dreuw, A.; Dunietz, B. D.; Furlani, T. R.; Goddard, W. A.; Hammes-Schiffer, S.; Head-Gordon, T.; Hehre, W. J.; Hsu, C.-P.; Jagau, T.-C.; Jung, Y.; Klamt, A.; Kong, J.; Lambrecht, D. S.; Liang, W.; Mayhall, N. J.; McCurdy, C.

W.; Neaton, J. B.; Ochsenfeld, C.; Parkhill, J. A.; Peverati, R.; Rassolov, V. A.; Shao, Y.; Slipchenko, L. V.; Stauch, T.; Steele, R. P.; Subotnik, J. E.; Thom, A. J. W.; Tkatchenko, A.; Truhlar, D. G.; Van Voorhis, T.; Wesolowski, T. A.; Whaley, K. B.; Woodcock, H. L.; Zimmerman, P. M.; Faraji, S.; Gill, P. M. W.; Head-Gordon, M.; Herbert, J. M.; Krylov, A. I. Software for the Frontiers of Quantum Chemistry: An Overview of Developments in the Q-Chem 5 Package. *J Chem Phys* **2021**, *155* (8), 084801. https://doi.org/10.1063/5.0055522.

(7) Plasser, F.; Krylov, A. I.; Dreuw, A. Libwfa: Wavefunction Analysis Tools for Excited and Open-shell Electronic States. *WIREs Comp Mol Sci* **2022**, *12* (4), e1595. https://doi.org/10.1002/wcms.1595.

(8) Frisch, M. J.; et al. Gaussian 16. Gaussian, Inc.: Wallingford 2016.

(9) Hättig, C.; Weigend, F. CC2 Excitation Energy Calculations on Large Molecules Using the Resolution of the Identity Approximation. *J Chem Phys* **2000**, *113*, 5154–5161. https://doi.org/10.1063/1.1290013.

(10) Balasubramani, S. G.; Chen, G. P.; Coriani, S.; Diedenhofen, M.; Frank, M. S.; Franzke, Y. J.; Furche, F.; Grotjahn, R.; Harding, M. E.; Hättig, C.; Hellweg, A.; Helmich-Paris, B.; Holzer, C.; Huniar, U.; Kaupp, M.; Marefat Khah, A.; Karbalaei Khani, S.; Müller, T.; Mack, F.; Nguyen, B. D.; Parker, S. M.; Perlt, E.; Rappoport, D.; Reiter, K.; Roy, S.; Rückert, M.; Schmitz, G.; Sierka, M.; Tapavicza, E.; Tew, D. P.; van Wüllen, C.; Voora, V. K.; Weigend, F.; Wodyński, A.; Yu, J. M. TURBOMOLE: Modular Program Suite for Ab Initio Quantum-Chemical and Condensed-Matter Simulations. *J Chem Phys* **2020**, *152* (18). https://doi.org/10.1063/5.0004635.
